# Glioma Cells With Genetically Engineered IGF-I Receptor Downregulation Can Persist in the Brain in a Dormant State

**DOI:** 10.3389/fonc.2020.555945

**Published:** 2020-09-23

**Authors:** Amir A. Samani, Josephine Nalbantoglu, Pnina Brodt

**Affiliations:** ^1^Department of Medicine, McGill University, Montreal, QC, Canada; ^2^Department of Neurology and Neurosurgery, Montreal Neurological Institute, McGill University, Montreal, QC, Canada; ^3^Department of Surgery, McGill University, Montreal, QC, Canada; ^4^Department of Oncology, McGill University, Montreal, QC, Canada; ^5^The Research Institute of the McGill University Health Center, Montreal, QC, Canada

**Keywords:** glioma, gene therapy, IGF, dormancy, signaling

## Abstract

Glioblastoma multiforme is an aggressive malignancy, resistant to standard treatment modalities and associated with poor prognosis. We analyzed the role of the IGF system in intracerebral glioma growth using human and rat glioma cells. The glioma cells C6 and U87MG were transduced with a genetically engineered retrovirus expressing type 1 insulin-like growth factor (IGF-IR) antisense RNA, either before or after intra-cerebral implantation of the cells into Sprague Dawley rats or nude mice, respectively and tumor growth and animal survival were monitored. Rat glioma cells transduced prior to orthotopic, intra-cerebral implantation had a significantly increased apoptotic rate *in vivo* and a significantly reduced tumor volume as seen 24 days post implantation (*p* < 0.0015). This resulted in increased survival, as greater than 70% of the rats were still alive 182 days after tumor implantation (*p* < 0.01), as compared to 80% mortality by day 24 in the control group. Histomorphology and histochemical studies performed on brain tissue that was obtained from rats that survived for 182 days revealed numerous single cells that were widely disseminated throughout the brain. These cells expressed the β-galactosidase marker protein, but were Ki67negative, suggesting that they acquired a dormant phenotype. Direct targeting of the C6 cells with retroviral particles *in vivo* was effective and reduced tumor volumes by 22% relative to controls. A significant effect on tumor growth was also seen with human glioma U87MG cells that were virally transduced and implanted intra-cerebrally in nude mice. We observed in these mice a significant reduction in tumor volumes and 70% of the animals were still alive 6 months after tumor implantation, as compared to 100% mortality in the control group by day 63. Our results show that IGF-IR targeting can inhibit the intracerebral growth of glioma cells. They also suggest that IGF-IR expression levels may determine a delicate balance between glioma cell growth, death and the acquisition of a dormant state in the brain.

## Introduction

Glioblastoma multiforme (GBM) is the most common neuroectodermal tumor and the most malignant of cerebral astrocytic gliomas. Despite multimodal treatment regimens, the prognosis for the majority of glioblastoma patients remains poor, with a median survival of less than 1 year (1),. There is therefore an urgent need for new therapeutic approaches.

The receptor for the type 1 insulin-like growth factor (IGF-IR) has been implicated in the acquisition of the transformed phenotype and identified as a positive regulator of cancer cell survival, growth and metastasis in a range of tumor types [reviewed in (2, 3)]. In many human malignancies, including GBM, upregulated expression of IGF-IR, IGF-I, IGF-II or combinations thereof have been documented (2, 4). Therefore, targeting the IGF system, by inhibiting ligand or receptor synthesis and/or function could provide effective therapeutic approaches to the treatment of GBM (5–9).

Viral vectors have generally been the vehicles of choice for the delivery of genetic information into tumor cells. Retrovirus-based vectors integrate selectively into actively dividing cells and are thus particularly suited for cancer gene therapy. Pseudotyped viral particles such as VSV-G expressing retro and lentiviruses were used effectively for gene delivery *in vivo* (10, 11).

We produced and evaluated a replication defective MMLV retroviral vector (vLTR-IGF-IR^AS^) in which an IGF-IR antisense fragment was expressed in a bi-cistronic RNA with EGFP, as we previously described (12). We assessed the anti-tumorigenic potential of this vector using orthotopically implanted human U87MG-LacZ and rat C6-LacZ cells. We found that in addition to causing extensive apoptosis, the downregulation of IGF-IR expression also induced a state of prolonged growth arrest in some of these, otherwise highly aggressive, glioma cells. The results suggest that IGF-IR levels in these cells may regulate a balance between cell growth, apoptosis and the acquisition of a dormant state.

## Materials and Methods

### Cell Lines

Rat glioma cell line C6/LacZ was from American Type Culture Collection (ATCC). The human glioma cell line U87 MG was obtained from the ATCC and transfected with a β-galactosidase expressing plasmid as previously described (13). The 293GPG retroviral packaging cell line (14) and the pLTR-GFP cells were maintained as previously described (15). All cells were routinely tested for mycoplasma and common rodent pathogens.

### Production of Retroviral Particles and Viral Transduction

The protocol used to construct the pLTR-IGF-IR^AS^ retrovector was previously described in detail (12). Retroviral particles were produced in the 293GPG packaging cell line and tittered as previously described (16). The pLTR-GFP packaging cell line was used to produce the control viral particles that express the EGFP gene only, using the same protocol. To virally transduce the C6/LacZ cells, 2–4 × 10^4^ cells/well were plated in a 6-well plate and after an overnight culture, 4 × 10^4^ vLTR-IGF-IR^AS^ or control pLTR-GFP retroviral particles were added per well on two consecutive days. The transduced cells were then sorted using a fluorescence activated cell sorter (FACSVantage, Becton Dickinson) and highly fluorescent cells (5–10% highest fluorescent intensity) were selected, designated C6/LacZ^AS^ and C6/LacZ^GFP^, respectively, and used for all the experiments. The same strategy was used to generate U87MG-LacZ^AS^ and U87MG-LacZ^GFP^ cells.

### RT-PCR Analysis

Five microgram of total RNA from each cell line were reverse transcribed using a primer corresponding to nt488-464 of mouse IGF-IR mRNA (100% homology; GeneBank accession # AF056187). PCR was performed using the same primer and a primer corresponding to nt89-113 of mouse IGF-IR. The cDNA products were electrophoresed on a 1% agarose gel, and the bands analyzed by densitometry using the ALPHAImager 2000 software (Alpha Innotech Corporation, San Leandro, CA, United States). The L19 signal was used to normalize the data (17).

### Western Blotting

Type 1 insulin-like growth factor receptor levels were analyzed by Western blotting performed as described in detail elsewhere (12) and using a rabbit anti-IGF-IR antiserum (C-20, Santa Cruz Biotechnology, Dallas, TX, United States) and a horseradish peroxidase-conjugated donkey anti rabbit IgG antibody (GE Healthcare Life Sciences, Pittsburgh, PA, United States). To normalize for loading, the membranes were stripped and re-probed with a monoclonal anti-tyrosine tubulin antibody (Sigma-Aldrich, St. Louis, MO, United States). To analyze ERK1 and 2 phosphorylation in response to IGF-I, tumor cells, cultured overnight in serum-free medium, were stimulated with 100 ng/ml IGF-I (US Biological, Salem, MA, United States) for 10 min, lysed on ice in the presence of phosphatase inhibitors and the cell lysates separated on 8.5% SDS-polyacrylamide gels. The blots were probed, first with an anti-phospho-p44/42 ERK (Thr202/Tyr204) antibody and then with an anti p44/42 ERK antibody (Cell Signaling, Whitby, ON, Canada).

### Functional *in vitro* Assays for IGF-I Responsiveness

Insulin-like growth factor-I induced cell proliferation was measured by the MTT [3-(4,5-Dimethylthiazol-2-yl)-2,5-diphenyltetrazolium bromide Thiazolyl blue] assay and cell survival in serum-depleted medium was measured using propidium iodide (PI) staining (12, 18).

### Tumor Cell Growth in Three-Dimensional (3D) Spheroids

Anchorage-independent tumor cell growth was analyzed using spheroids generated from hanging drops, as was previously described (19). Briefly, confluent cultures of C6 cells in 10% FCS DMEM were dispersed by trypsin digestion and the cells resuspended in DMEM. Twenty microliter drops containing viable cells were placed on the lids of 100 mm culture dishes, which were then inverted over dishes containing 10 ml DMEM and incubated at 37°C for 30 days. The cellular aggregates were then harvested using a Pasteur pipette under a dissecting microscope, placed in 100 mm culture dishes pre-coated with 0.75% agar and overlaid with 10 ml DMEM. The spheroid surface areas were calculated using an inverted microscope equipped with an ocular grid.

### Brain Tumor Growth and Apoptosis *in vivo*

All animal experiments were performed in strict adherence to the McGill University Animal Care committee guidelines. Tumor cell implantation was performed as previously reported (13, 16). Briefly, 10^4^ rat, or 10^5^ human glioma cells were injected intra-cranially into the caudate of adult Sprague Dawley rats or nude mice, respectively, in a stereotactic apparatus (Kopf, Tujunga, CA, United States). Tumor volume measurements were performed on coronal (10 μm) sections stained histochemically for β-galactosidase activity, as described, and the volumes calculated as length (mm) × the square of the width (mm)^2^ × 0.4. For long-term survival studies, the animals were monitored from the day of tumor implantation until the onset of morbidity and euthanized, as per the McGill University’s guidelines for animal care. Apoptotic cells were visualized based on the incorporation of biotinylated dUTP into nicked DNA that was detected by incubation with Cy3-streptavidin (Jackson Lab).

### Delivery of Viral Particles *in vivo*

C6/LacZ cells were implanted as described above. Six days later, rats were anesthetized and vLTR-IGF-IR^AS^ or vLTR-GFP (concentrated stock of 3 × 10^8^ cfu/ml) were injected into six different sites (1 mm apart) in the pre-established tumor, guided by the previous stereotactic coordinates. A total volume of 9 μl were injected into each tumor (6 × 1.5 μl increments), and the needle left in place for at least 5 min/increment (for a total of 30 min/tumor). This procedure was repeated one week later. Rats were euthanized 8 days after the last injection. Fluorescence microscopy was used to detect GFP expression in frozen brain sections. Subsequently, sections were stained histochemically for β-galactosidase activity and tumor volumes calculated based on these measurements.

### Immunofluorescence

Cryostat sections were air-dried for 30 min, fixed with 4% paraformaldehyde and non-specific staining blocked using 5% normal serum derived from the same species as the secondary antibody. The sections were then incubated overnight at 4°C with the primary antibodies at dilutions of 1:10 (mouse monoclonal anti-Ki-67, Chemicon, Temecula, CA, United States) or 1:100 (rabbit-anti-IGF-IR, Santa Cruz, Santa Cruz, CA, United States), FITC conjugated rabbit anti-β-galactosidase (Rockland, Gilberstville, PA, United States) or mouse monoclonal anti-GFP (Chemicon, Temecual, CA, United States) all in PBS containing 0.1% Trition X-100, 0.1% BSA and 0.1% normal serum.

This was followed by a 1 h incubation at RT with Alexa Fluor 568 goat anti-mouse IgG (Molecular Probes, Burlington, ON, Canada) for detection of Ki-67 and GFP or with Alexa Fluor 488 goat anti-rabbit IgG (Molecular Probes, Eugene, OR, United States) and Cy-3 conjugated donkey anti-rabbit IgG (Jackson Immunoresearch, West Grove, PA, United States) for detection of IGF-IR, all at a dilution of 1:200.

### Statistical Analysis

The one or two-sided Student’s *t*-test were used to analyze differences observed in tumor volumes or in functional *in vitro* assays, respectively. The Kaplan–Meier survival curve and the Log-Rank test were used with Statistica software to analyze survival data.

## Results

### Enhanced IGF-IR Expression at the Invasive Margins of C6 Tumors Growing in the Brain

**FIGURE 1:**
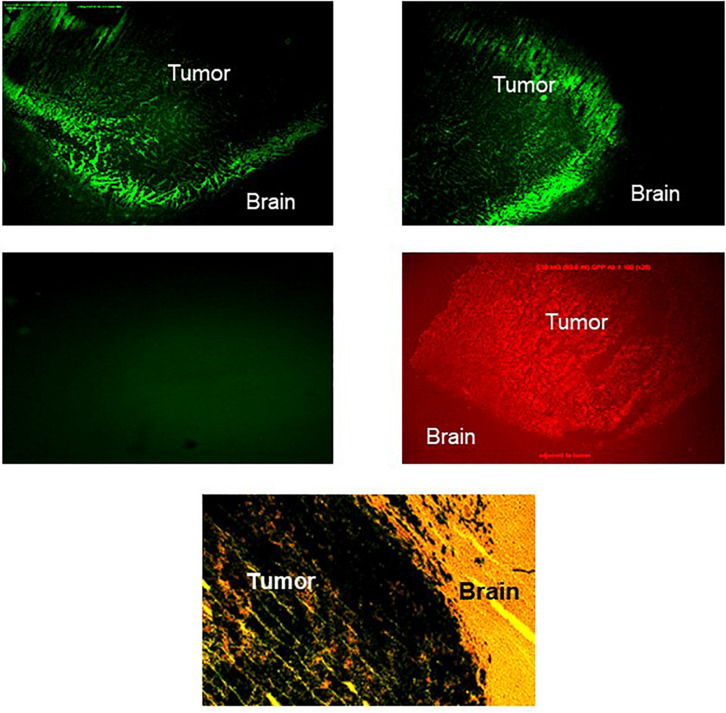
Increased IGF-IR expression in the invasive margins of intra-cerebral rat glioma implants. Brain sections were prepared 20 days after the intra-cranial implantation of 10^4^ C6 cells. The tissue was processed and immunostained as described in the section “Materials and Methods,” using anti IGF-IR (**top panel, left** and **right**, showing two different areas of the tumor-brain margin) or anti-GFP **(middle panel, right)** antibodies. Control sections were incubated with goat anti rabbit IgG antibody only **(middle panel, left)**. The green emission background of the GFP positive glioma cells in all sections was minimized by lowering the exposure time (see **middle panel, left**). A parallel section stained for β-galactosidase activity and counter stained with hematoxylin and eosin is shown on the bottom (×120 magnification).

We first assessed the expression of IGF-IR in GFP^+^ C6 glioma cells that were implanted into the caudate region of the rat brain, using IHC. IGF-IR expressing cells were observed throughout the tumor area. However, the intensity of fluorescence was highest at the invading margins of the tumors, underlining the preferential localization of IGF-IR-expressing cells at sites of active invasion (Figure 1). In contrast to the region-specific pattern of IGF-IR expression, GFP staining was uniform throughout the tumor mass (Figure 1), indicating that the increased intensity of IGF-IR at the tumor margins was not due to a greater concentration of viable cells in this region, or to increased cell death in the inner mass of the tumor. To inhibit IGF-IR signaling during intracerebral tumor growth, we then used a retroviral vector expressing IGF-IR antisense to downregulate receptor expression.

### Reduced IGF-IR Levels, Suppressed IGF-I Responses and Impaired Intra-Cerebral Growth of C6 Cells Transduced With vLTR-IGF-IR^AS^ Particles

**FIGURE 2:**
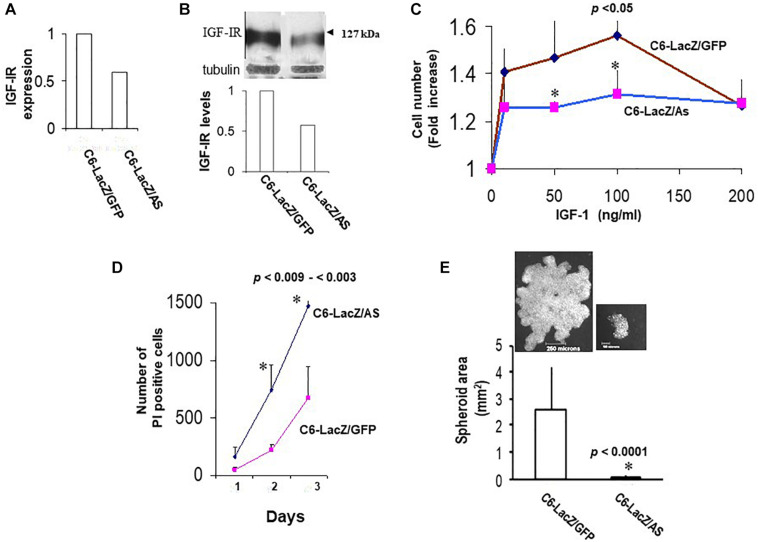
Reduced IGF-IR expression in virally transduced C6 cells affects multiple IGF-I – regulated functions. IGF-IR mRNA expression levels **(A)** were measured by RT-PCR. Representative results of densitometry analysis (*n* = 2) shown in the bar graph are expressed as the ratios of the densities of IGF-IR:L19 bands relative to control C6/LacZ^GFP^ cells that were assigned a value of 1. Protein levels **(B)** were analyzed by Western blotting. Representative results of laser densitometry, shown in the bar graph (*n* = 2) are expressed as the ratios of IGF-IR:tubulin bands relative to C6/LacZ^GFP^ cells that were assigned a value of 1. Cell proliferation **(C)** was measured by the MTT assay. Shown are the results of a representative experiment of 3 performed. Data are expressed as the means and SD of triplicate samples (^∗^*p* < 0.05 for 50 and 100 ng/ml IGF-I). Cell survival in IGF-I containing medium **(D)** was analyzed by PI staining. Glioma cells were plated in 4-chamber slides, first in complete medium and then in serum-free RPMI medium containing 100 ng/ml rhIGF-I. PI (1 mg/ml) was added to the chambers for 5 min at 37°C, on days 1–3 and the PI-permissible (dead) cells enumerated using an inverted microscope. Data are expressed as means (±SD) of triplicate samples (^∗^*p* < 0.003 and *p* < 0.009 for days 2 and 3, respectively). Shown in **(E)** are means (±SD) of total spheroid surface area for the indicated cells. Images of representative spheroids for each of the cells are shown on top.

Sublines C6/LacZ^AS^ and C6/LacZ^GFP^ were generated by transduction of C6/LacZ cells with vLTR-IGF-IR-antisense (vLTR-IGF-IR^AS^) and vLTR-GFP (vLTR-GFP)-expressing retroviral particles, respectively. Gene transfer efficiency in these cells was 45 and 80%, respectively as revealed by flow cytometry analysis. Highly fluorescent C6/LacZ^AS^ cells (top 5–10 percentile) were enriched by FACS sorting to yield a population in which 77% of the cells were highly GFP – positive. PCR and Western blotting confirmed reductions of 40 and 42% in mRNA (Figure 2A) and protein (Figure 2B) levels in these cells, respectively, as compared to control, C6/LacZ^GFP^ cells. The reduction in IGF-IR expression levels resulted in a markedly decreased IGF-I responsiveness in these cells, as reflected in reduced cell proliferation revealed by the MTT assay (up to 53% reduction, Figure 2C), increased apoptosis in serum depleted medium supplemented with IGF-I, as measured by PI staining (2.2-fold increase in PI positive cells, Figure 2D), and reduced anchorage-independent growth, as assessed in a 3D spheroid assay (Figure 2E).

**FIGURE 3:**
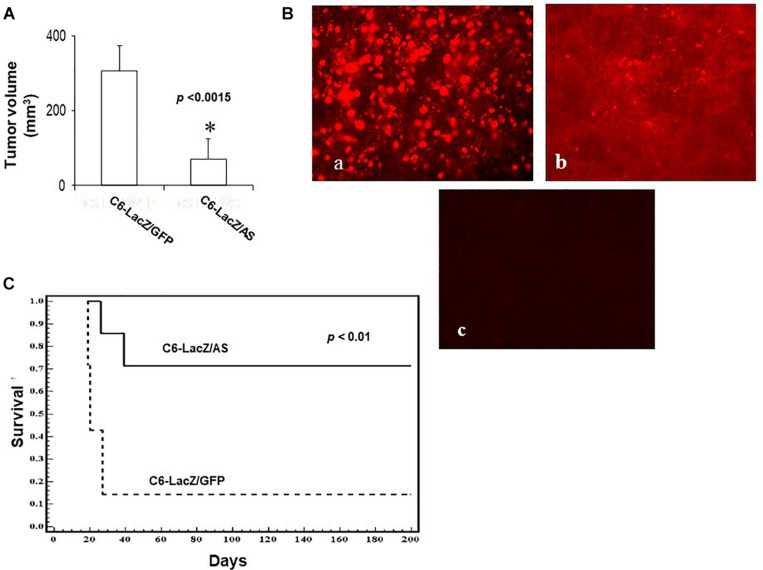
Reduced intracerebral growth of C6/LacZ^AS^ cells. Rats were implanted with 10^4^ cells intra-cranially and the brains removed for tumor volume measurement as described in section “Materials and Methods.” Shown in **(A)** are the results of a representative experiment of two performed (*n* = 5 in each, ^∗^*p* < 0.0015). Shown in **(B)** are results of a TUNEL assay performed on representative sections. Apoptotic cells were visualized using Cy3-conjugated streptavidin (**a**-C6/LacZ^AS^; **b**-C6/LacZ^GFP^). Control sections **(c)** prepared from C6/LacZ^AS^ injected animals were treated in the same manner but without the terminal deoxynucleotidyl transferase (TdT) enzyme. Long-term survival of rats implanted intra-cranially with 10^4^ C6/LacZ glioma cells **(C)** was analyzed in three independent experiments using 9–14 animals per group. The animals were followed until the onset of morbidity, at which point they were euthanized. Disease-free rats were euthanized 182 days post tumor implantation (^∗^*p* < 0.01).

The reduction in IGF-IR-mediated functions had a marked effect on glioma cell tumorigenicity in the brain, following intra-cerebral implantation. While 80% of the rats injected with C6/LacZ^GFP^ cells were moribund by 24 days following intra-cerebral tumor implantation, all the rats in the C6/LacZ^AS^-injected group were still alive at that time. Tumor volume measurements revealed that although C6/LacZ^AS^ cells could initially form small tumors, their ability to expand in the brain and produce large tumor masses was significantly impaired (*p* < 0.0007) relative to the controls (Figure 3A). A TUNEL assay performed on brain sections showed the presence of numerous apoptotic cells in the C6/LacZ^AS^-injected rats as compared to no or few TUNEL-positive cells in the controls (Figure 3B), confirming that reduced tumor growth in the brain resulted from increased apoptosis in these cells. No significant differences in tumor volumes or the proportions of TUNEL-positive cells were observed when C6/LacZ and C6/LacZ^GFP^ tumors were compared. The profound effect that IGF-IR downregulation had on the growth of these cells *in vivo* was akin to the significant growth reduction observed in the spheroid assay (Figure 2E), consistent with results reported by others on the importance of IGF-IR for anchorage independent 3D tumor growth (20, 21).

When the effect of reduced IGF-R expression in the tumor cells on rat survival was subsequently analyzed, we found a significant increase in long-term survival of rats inoculated with C6/LacZ^AS^, as compared to controls. Namely, while 86% of the rats injected with C6/LacZ^GFP^ cells were moribund by day 24 following tumor cell inoculation, 72% of the rats injected with C6/LacZ^AS^ cells were clinically disease-free until day 182, at which time they were euthanized (*p* < 0.01, Figure 3C).

### Solitary, Growth Arrested C6/LacZ^AS^ Cells Can Be Detected in the Brains of C6/LacZ^AS^ Injected Rats Several Months After Tumor Implantation

**FIGURE 4:**
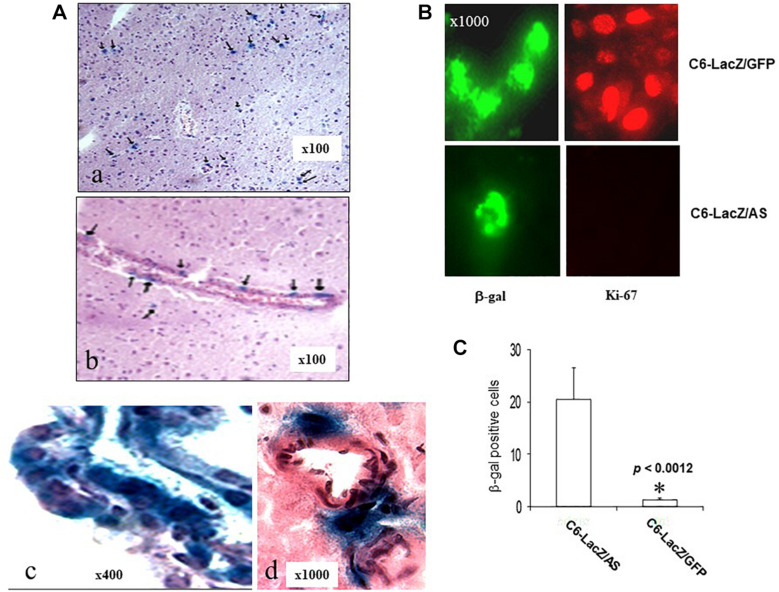
Long-term survival of solitary, intra-cerebral tumor cells in C6/LacZ^AS^-injected rats. Brain sections (10 μm) of rats that survived 182 days following C6-LacZ^AS^ implantation **(A)** were immunostained to detect β-galactosidase activity. Shown are solitary cells identified in the brain parenchyma **(a)**, adjacent to ventricles **(b)**, within the choroid plexus **(c)** and in the perivascular space associated with small arterial vessels **(d)**. Immunohistochemistry **(B)** was performed on sections derived from the indicated surviving rats using antibodies to Ki-67 (right) or β-galactosidase (left). LacZ positive cells in the sections were quantified **(C)** in five random fields (×100 magnification) per section and three sections were analyzed per brain. Shown are mean numbers of β-gal^+^ cells (±SD) in a total of 10–15 sections analyzed per group. ^∗^*p* < 0.0012.

In brain sections prepared from clinically disease-free C6/LacZ^AS^-injected rats, 182 days after tumor injection, we identified single LacZ positive cells in ipsilateral and/or contralateral regions of the brain (Figure 4A). In some animals, they were also seen around the choroid plexus in the ventricles (Figure 4A, bottom). These cells could also be detected by immunofluorescence staining using an FITC-conjugated rabbit anti β-galactosidase antibody (Figure 4B). Cells in all regions were solitary and no foci could be seen in a total of 150 sections (15 sections/rat, *n* = 10) examined. The robust expression of β-galactosidase indicated that the glioma cells were alive until the end of the experiment. However, no Ki-67 staining could be detected in these cells, indicating that they were in cell cycle arrest (Figure 4B). Two of the 10 rats injected with the C6/LacZ^GFP^ cells also survived for 6 months. We therefore used them to compare the incidence of growth-arrested solitary tumor cells in the two groups. Analysis of three sections/rat derived from different regions of the brain revealed that in animals injected with C6/LacZ^AS^ cells, the number of solitary LacZ^+^ cells was 20-fold greater than in C6/LacZ^GFP^ injected rats (Figure 4C). This suggested that the acquisition of a prolonged state of cell cycle arrest by C6/LacZ^AS^ cells was not random but rather a specific outcome of reduced IGF-IR expression in these cells.

### Direct Intra-Cerebral Inoculation of vLTR-IGF-IR^AS^ Particles Also Inhibits Tumor Growth

**FIGURE 5:**
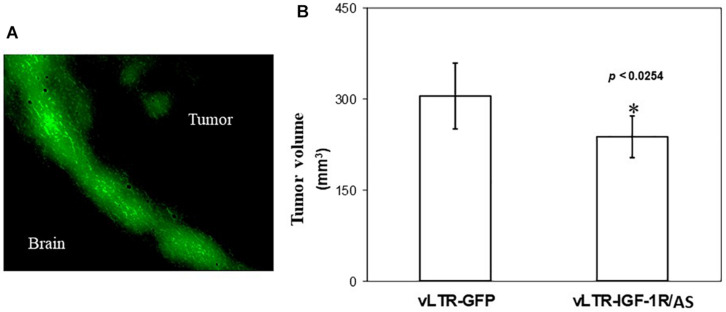
*In vivo* targeting of pre-implanted rat glioma cells with IGF-IR antisense expressing viral particles reduces tumor expansion. Shown in **(A)** are intra-cerebral C6 cells following *in vivo* transduction with vLTR-GFP retroviral particles. GFP-expressing cells were observed mainly at the expanding edge of the tumors. Shown in **(B)** are mean tumor volumes (±SD) following two injections of vLTR-IGF-IR^AS^ or control vLTR-GFP particles, 6 and 13 days post orthotopic implantation of C6/LacZ cells (*n* = 5, ^∗^*p* < 0.0254).

In order to determine the ability of vLTR-IGF-IR^AS^ to target pre-established tumors growing in the brain, rats were injected intra-cranially with 2.7 × 10^6^ vLTR-IGF-IR^AS^ or vLTR-GFP particles, 6 and 13 days following the orthotopic implantation of the C6/LacZ cells. Fluorescence microscopy confirmed the presence of GFP^+^ C6/LacZ cells, localized mainly at the advancing edge of the tumors (Figure 5A), indicating that the retroviral particles were able to transduce cells within pre-established C6/LacZ brain tumors. All the animals were euthanized 24 days post tumor implantation and tumor volumes were calculated. We found a 22% reduction in the mean tumor volume in rats inoculated with vLTR-IGF-IR^AS^ as compared to controls injected with the vLTR-GFP particles (*p* < 0.026, Figure 5B). Importantly, while 40% of the control rats were already moribund at this time, no morbidity was observed in the vLTR-IGF-IR^AS^ – injected group.

### Human U87 Glioma Cells Transduced With vLTR-IGF-IR^AS^ Particles Also Have Reduced Growth *in vivo*

**FIGURE 6:**
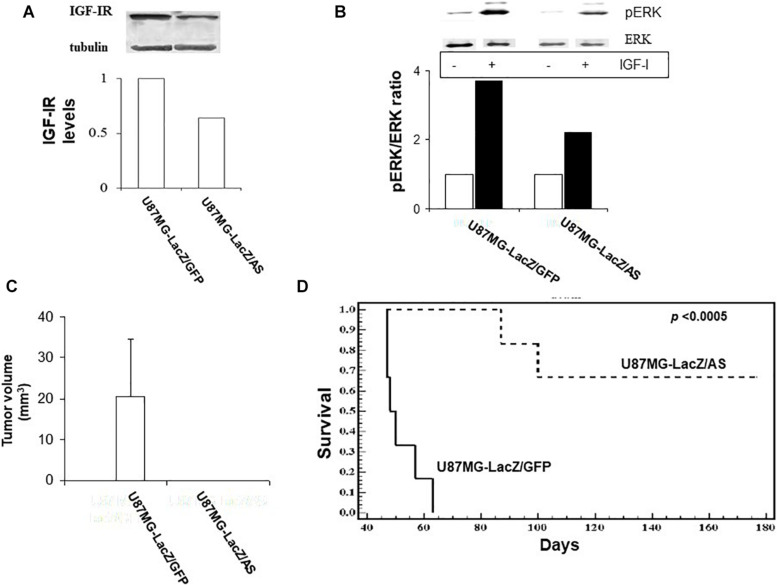
Reduced IGF-IR expression in virally transduced U87MG cells inhibits IGF-I responsiveness *in vitro* and tumor growth *in vivo*. U87 cells were transduced with vLTR-IGF-IR^AS^ or vLTR-GFP particles. IGF-IR levels were analyzed by Western blotting **(A)**. Shown are the results of a representative blot (*n* = 2). Results of the laser densitometry, shown in the bar graph are expressed as the ratios of IGF-IR:tubulin relative to U87MG-LacZ/GFP cells which were assigned a value of 1. ERK phosphorylation **(B)** was measured following the incubation of serum-starved tumor cells with 100 ng/ml IGF-I for 10 min. Blots were probed consecutively with antibodies to phospho-p44/42 ERK and p44/42 ERK. Shown are the results of one representative experiment of two performed. Results in the bar graph are expressed as mean increase in pERK:ERK ratios relative to control, untreated cells (analyzed in the same gel) that were assigned a value of 1 (*n* = 2). Nude mice were injected intra-cranially with 10^5^ U87 cells as indicated. Tumor volumes **(C)** were measured on day 35 post injection and survival **(D)** was analyzed in separate groups of mice injected as above. Shown in **(C,D)** are pooled data from two separate experiments, each performed with 6 nu/nu mice per group. ^∗^*p* < 0.0005.

We subsequently investigated the effect of retrovirally induced suppression of IGF-IR expression on the growth of human glioma cells, using the U87MG cell line. Sublines U87MG-LacZ/AS and U87MG-LacZ/GFP were generated as described for C6 cells. The efficiency of gene transfer in these cells, as determined by flow cytometry was 48 and 94%, respectively. Western blotting revealed a 36% reduction in IGF-IR expression in U87MGLacZ/AS cells relative to controls (Figure 6A). This resulted in reduced IGF-IR signaling, as reflected in a 40% reduction in IGF-I induced ERK activation in these cells, as compared to control, U87MG-LacZ/GFP cells (Figure 6B).

The effects of IGF-IR suppression on the intra-cerebral growth of U87MG cells were more profound than those observed in the rat glioma model. In a tumor volume study (Figure 6C), all U87MG-LacZ/GFP–implanted, but none of the U87MG-LacZ/AS implanted mice had sizable tumors by day 35 post implantation. This was reflected in a significant difference in long-term survival because all mice implanted with U87MG-LacZ/GFP were euthanized for neurological symptoms by day 63 post implantation, while 70% of those implanted with U87MG-LacZ/AS cells were clinically disease free until day 176, at which time they were euthanized (Figure 6D). Interestingly, in nude mice implanted with U87MG-LacZ/AS cells, the presence of solitary β-gal^+^ tumor cells in the brains was limited to the injection tracts (not shown) and could only be seen in mice sacrificed by day 35, when tumor volumes were measured, suggesting that reduced IGF-IR levels eventually caused the death of all U87MG cells.

## Discussion

The critical role that the IGF axis plays in the growth of GBM has been well documented in various experimental models (6, 22, 23). In early studies, the IGFs were shown to enhance 3D growth of glioblastoma (21) and more recently, high IGF-IR expression levels were identified as an independent prognostic factor associated with shorter survival, a poorer response to temozolomide (4) and resistance to anti-EGFR (24) and anti-PDGFR (25) treatments in GBM patients. Recently, it was shown that elevated expression of IGF-IR and IGF-II in GBM were associated with poor patient survival and that paracrine IGF-IR/IGF-II signaling promoted the expansion of a chemoresistant glioma subpopulation (26). The levels and patterns of IGF expression were also shown to correlate with histopathologic grade in diffusely infiltrating astrocytomas (27).

In the brain, glioma cells can respond to IGF ligands originating from several potential cellular sources. They may produce the factor and utilize it in an autocrine fashion (26, 28), or they could utilize IGF-I produced by reactive astrocytes or microvascular cells exhibiting endothelial/pericytic hyperplasia at the margins of tumor infiltration, as was shown by Hirano et al. (27). Interestingly, while in normal cells, IGF-I typically binds to IGF-IR with (5–10-fold) higher affinity than IGF-II, glioma cells were shown to express a receptor with a unique IGF-IRα subunit of higher molecular size that has similar affinities for both ligands (29). This provides the tumor cells with a unique advantage, because they are able to respond more effectively to IGF-II that is produced at high concentrations in the brain (30, 31). This dual, high affinity binding may also limit the efficacy of therapeutic strategies designed to target individual ligands (32) and underlines the importance of targeting the receptor (9, 32) or both ligands. Targeting IGF-IR expression using strategies such as gene therapy also has the added advantage of reducing the levels of receptor available to translocate to the nucleus, where it was shown to play a regulatory role, independent of its membrane-bound functions (33).

The critical role of the IGF axis in glioma progression has been confirmed in multiple studies including by Resnikoff et al. (34) who used an antisense strategy to silence IGF-IR expression in C6 cells. However, few studies have actually examined the effects of reduced IGF-IR expression, or function, on glioma cell growth and spread orthotopically, in the brain (7, 35). Using the C6 cells, we document here high expression of IGF-IR at the invasive margins of expanding tumors, suggesting that glioma cells in the margins of expanding tumors are uniquely positioned to respond to IGF ligands produced by the microenvironment, an interaction also documented in a recent study by Quail et al. (36). Interestingly, high IGF-IR levels were also documented at the invasive margins of other tumors such as colorectal carcinoma liver metastases, where they were shown to coincide with high IGF-II expression in adjacent, hepatic stromal cells (37).

Viral particles have been employed by others to deliver antisense RNA into tumor cells. This approach was used to target oncogenes such as K-Ras (38) and c-fos (39, 40), growth and survival factors such as IGF-I (41), TGF-β (42) and bcl-2 (43) and, more recently, telomerases (44). The present work adds to this body of evidence and shows that growth factor receptor-targeting by antisense RNA delivered via VSVG-modified viral particles can affect the tumorigenic phenotype of the cells when delivered intra-tumorally *in vivo*. The results also establish the feasibility of obtaining effective levels of suppression for the antisense-targeted gene, while maintaining high expression levels for a second, reporter gene such as EGFP coexpressed in a bi-cistronic transcript.

Our data show that a reduction in IGF-IR expression could affect intra-cerebral glioma growth in one of several ways. While the majority of C6/LacZ^AS^ cells became apoptotic by day 24 following injection, others survived, and either continued to proliferate, eventually leading to morbidity, or they entered a prolonged state of growth arrest as solitary cells. While the mechanisms determining ultimate tumor cell fate in our model remain to be fully elucidated, several possibilities can be proposed. The C6/LacZ^AS^ and U87MG-LacZ/AS cells used in this study were polyclonal, consisting of cells with variable IGF-IR expression levels that could affect the susceptibility to, and outcome of antisense-mediated suppression. Moreover, the transgene expression levels were also variable as reflected in a range of GFP levels in these cells. Although the use of a heterogeneous population can potentially lead to the range of responses that we observed, it was our preferred approach because it more closely mimics the potential effects of direct viral transduction in a clinical setting. In this context, it is relevant that a threshold effect was previously documented for IGF-IR-mediated signaling and function. Namely, in embryonic fibroblasts derived from IGF-IR - null mice ectopically expressing IGF-IR, a requirement for a threshold of 1.5 × 10^4^ receptors per cell was demonstrated for IGF-I-induced DNA synthesis and of 2.2 × 10^4^ and 3 × 10^4^ ligand-binding sites per cell, respectively, for cell proliferation and transformation (45, 46). Similar observations were reported in a fibrosarcoma model (47) and in our own studies with a Lewis lung carcinoma model (48). Thus, C6 cells may have become apoptotic if their IGF-IR expression levels were below the threshold required for survival, or entered cell cycle arrest if they expressed the requisite receptor levels for survival but not for cell cycle entry. A variable response of the tumor microenvironment to tumor cells with different IGF-IR expression levels may also have contributed to the divergent outcomes, as IGF-IR signaling levels were shown to affect tumor cell immunogenicity (23, 34).

The latter finding was, in fact, the basis of a small clinical trial where immunization of glioma patients with autologous glioma cells expressing antisense IGF-IR had a beneficial effect, resulting in clinical and radiographic improvements in 8 of 12 patients, including three spontaneous or postsurgical regressions at either the primary or a distant intracranial site (49). Exosomes released by immunizing glioma cells were subsequently implicated in the immunizing effect. While we cannot at present rule out a contribution by the innate and/or adaptive immune response to the tumor inhibitory effect of IGF-IR silencing observed in our study, our finding of profound growth suppression of U87MG cells that were orthotopically implanted in nude mice suggest that it was not predicated on an intact T cell immune response. Of interest in this context is a recent study by Quail et al. (36), who found that in a transgenic mouse model of spontaneous GBM, cells that were dormant following colony stimulating factor receptor 1 (CSF-1R) inhibition became resistant to the treatment and were rescued from the dormant state by stroma-derived IGF-I, adding support for the role of IGF signaling in maintaining the balance between tumor cell proliferation, death and the acquisition of a dormant state. The reason for the prolonged survival of a small but detectable number of control C6/LacZ^GFP^ cells in a small number of control rats is presently unclear and may also be related to clonal heterogeneity in this tumor line.

The C6 cells are chemically induced rat brain cancer cells widely used as a glioma model for *in vitro* and *in vivo* studies (50). In recent years, the relevance of these cells as a model for human glioma has been questioned because their origin as chemically induced brain tumors may better represent “gliosarcomas” (51). It should be noted however, that in the present study, and in line with other studies (52), we did not observe the sarcomatous differentiation characteristic of gliosarcomas. Furthermore, in different studies, both the phenotype and immune microenvironment of experimental rat C6 gliomas were shown to resemble those of human glioblastoma (53, 54). Also of note, the clinical features and outcomes of gliosarcoma and glioblastoma, as revealed in clinical studies are essentially indistinguishable (55). Taken together with the results we obtained with the U87MG model, we believe that the present findings are of relevance to the management of human glioma. Of interest, loss of PTEN was identified as a resistance mechanism against IGF-targeting drugs in high grade gliomas (56) and other malignancies (57). Intriguingly, our results have shown that IGF-IR silencing was effective in PTEN-null U87MG cells (58). While the mechanism underlying the heightened sensitivity of these cells within the brain microenvironment remains to be elucidated, it is possible that the MEK/ERK pathway that is activated by IGF-I in these cells (as shown in Figure 6) determines their sensitivity to IGF-IR inhibition, in the presence of aberrant PI3K/PTEN/Akt signaling.

Type 1 insulin-like growth factor targeting may be highly beneficial in combination therapy with other anti-cancer drugs, because IGF-IR signaling was identified as a resistance mechanism for both chemo and targeted therapies (2). In particular, resistance to EGFR inhibition in human glioma cells was attributed to compensatory signaling by IGF-IR (59), and insulin receptor/IGF-IR signaling was shown to confer resistance to Gefitinib in EGFR-dependent glioblastoma, through compensatory AKT activation (24). Co-targeting of the IGF-IR and EGFR axes may therefore provide an efficacious therapeutic strategy for GBM (24). It may also be relevant to brain metastases originating from other malignancies, such as breast cancer (60).

## Conclusion

In conclusion, our data demonstrate that a gene therapy approach to IGF-IR silencing *in situ* holds promise as a strategy for limiting glioma growth *in vivo* either as monotherapy or in combination with other drugs. However, they also raise a cautionary note that a better understanding of the role of the IGF-IR in regulating the delicate balance between glioma cell death, dormancy and proliferation will be critical to the optimization of this approach and to effective control of residual disease.

## Data Availability Statement

The raw data supporting the conclusions of this article will be made available by the authors upon reasonable request, without undue reservation.

## Ethics Statement

The animal study was reviewed and approved by the McGill University Animal Care Committee.

## Author Contributions

AAS planned, performed, and analyzed all the experiments and drafted the manuscript. JN provided resources and guidance for the performance of animal experiments. PB oversaw the study, planned the experiments, and edited the manuscript. All authors contributed to the article and approved the submitted version.

## Conflict of Interest

The authors declare that the research was conducted in the absence of any commercial or financial relationships that could be construed as a potential conflict of interest.
